# A reliable quantitative method for determining CBD content and release from transdermal patches in Franz cells

**DOI:** 10.1002/pca.3188

**Published:** 2022-11-13

**Authors:** Liyun Yu, Frederikke Bahrt Madsen, Sofie Helvig Eriksen, Aaron J. C. Andersen, Anne Ladegaard Skov

**Affiliations:** ^1^ Danish Polymer Centre, Department of Chemical and Biochemical Engineering, Building 227 Technical University of Denmark Kgs. Lyngby Denmark; ^2^ Glysious Holte Denmark; ^3^ Department of Biotechnology and Biomedicine, Building 221 Technical University of Denmark Kgs. Lyngby Denmark

## Abstract

**Introduction:**

There are several cannabidiol (CBD) transdermal patches available on the market. However, none are FDA‐approved. Furthermore, not much evidence has been published about CBD release and skin permeation from such patches, so the effectiveness and reliability remain unclear.

**Objectives:**

We aimed to develop a method to determine the in vitro release and skin permeation of CBD from transdermal patches using Franz cell diffusion in combination with quantitative ^1^H‐NMR (qNMR).

**Materials and Methods:**

The study was conducted on CBD patches with known CBD content and six different commercially available or market‐ready CBD patches using a Franz cell with a Strat‐M™ membrane and with samples taken directly from the transdermal patch for qNMR analysis.

**Results:**

The use of qNMR yielded an average recovery of 100% ± 7% when samples with known CBD content were tested. Results from the testing of six commercially available patches indicated that five out of six patches did not contain the CBD amount stated by the manufacturer according to a ± 10% variance margin, of which four patches were under‐labeled and one was over‐labeled. The release rate of patches was determined, and significant differences between the patches were shown. Maximum release of CBD was calculated to occur after 39 to 70 h.

**Conclusion:**

The established method was proven to be a reliable means of determining the quantity and release of CBD from transdermal patches and can be used to verify CBD content and release rate in transdermal patches.

## Abstract 2

We describe the use of Franz cell diffusion in combination with quantitative 1H‐NMR (qNMR) as a reliable method to determine the in vitro release and skin permeation of CBD from transdermal patches. Results from testing commercially available patches proved that 5 out of 6 patches did not contain the CBD amount stated by the manufacturer according to a ±10% variance margin wherein four patches were under‐labeled, and one was over‐labeled.

## 1 INTRODUCTION

Cannabidiol (CBD) is one of the many cannabinoids in the cannabis plant. In recent years, CBD has attracted increasing interest both in commercial products and in the scientific community, with several scientific publications documenting the positive clinical effects of CBD. Contrary to another well‐known cannabinoid, Δ^9^‐tetrahydrocannabinol (THC), CBD is not psychoactive and thus has more potential in therapeutic applications. Clinical studies suggest that CBD has possible applications in treating neurological conditions such as epilepsy,^1^, ^2^ Alzheimer's disease,^3^ Parkinson's disease,^4^, ^5^ sleep disorders,^6^, ^7^ depression and psychotic disorders,^8^ anxiety,^8^ schizophrenia,^9^ and post‐traumatic stress disorder.^10^ CBD has also been shown to have positive dermatological effects such as anti‐inflammatory^11^, ^12^ and antioxidant effects,^13^ as well as in treating skin eczema, atopic dermatitis, acne vulgaris, and psoriasis.^11^, ^14^ CBD is furthermore commonly used in the treatment of acute and chronic pain.^15^, ^16^

CBD can be administered through oral, nasal, and transdermal/topical delivery. Oral delivery is the most conventional delivery approach, with some commercially available products on the market, including GW Pharmaceuticals FDA‐approved products Epidiolex® and Sativex®. Oral delivery has several limitations and drawbacks since the lipophilic nature of CBD negatively affects the solubility and hence bioavailability of the drug. Cannabinoid bioavailability and absorption via oral administration range from 6%^10^ to 20–30%.^12^ Nasal delivery may provide faster absorption and higher CBD bioavailability than oral administration, with reported values of 31%^10^ and 34–46%^17^ in animal studies. The lipophilic nature of CBD may, however, render crossing the nasal tissue difficult. Nasal delivery could also lead to irritation of the nasal mucosa and mucociliary clearance^15^ as well as increase the concentration of cannabinoids in the brain and consequently increase the risk of damage to the central nervous system in the case of residual amounts of psychoactive cannabinoids.^17^ This means that the nasal route is less suited for chronically administered drugs.^18^

Transdermal and topical delivery holds some advantages over oral delivery since first‐pass metabolism, where the concentration of CBD gets significantly reduced, is avoided along with the degradation of CBD by enzymes in the gut. Topical delivery of CBD also provides a better safety profile since the plasma drug levels rise slowly.^17^ Transdermal delivery is a user‐friendly delivery system, allowing for higher patient compliance and thus more significant and robust clinical results.^19^, ^20^, ^21^

The lipophilic nature of CBD as well as its relatively low molecular weight (<500 Da) renders easy permeation through the hydrophobic outer epidermal barrier of the skin that is commonly blocking the permeation of drugs. Topical delivery, in the form of ointments, creams, gels, lotions, and patches, is used for local treatment of pain or skin conditions. In contrast, transdermal delivery is used when systemic effects are desired, and drugs are typically administered via transdermal patches.^22^ For transdermal delivery, penetration into the dermis and the inherent blood vessels is needed, and due to the complex structure of the aqueous dermis, this may not be straightforward. Several methods have been investigated to improve the transdermal delivery of CBD, including permeation enhancers, vesicles (drug carriers), liposomes, polymeric carriers, and physical permeation enhancers.^23^ However, there is still not much evidence about CBD's release and skin permeation from dermal patches. Thus, the effectiveness and reliability of, in particular, online‐bought CBD dermal patches remain unclear, and there are currently no FDA‐approved CBD patches on the market.

In vitro static diffusion cells are an essential tool in the assessment of skin permeability, indicating the bioavailability of the drug and effectiveness of the transdermal patch, as well as providing a method to evaluate the release properties of drugs from transdermal delivery media.^24^ Franz diffusion cells are commonly used for in vitro transdermal assessment and are named after their inventor, T. J. Franz, who developed the technique in 1975.^25^ The method mimics in vivo skin conditions and allows for an in vitro evaluation of the transdermal diffusion of skin care products. Franz concluded at that time that in vitro analyses using Franz diffusion cells correlate well with in vivo measurements, with the deviation depending on the permeability of the measured compound.^25^ The Franz diffusion cell system consists of a donor chamber, the Franz cell top in Figure 3, and a receptor chamber, the Franz cell, which are separated by a skin‐mimicking membrane. The medium is collected in the receptor chamber, from which samples can be taken out for sample analysis at different time intervals. The Franz cell is held at a constant temperature of 32°C to mimic skin conditions.

Franz cell diffusion has previously been used with methods such as high‐pressure liquid chromatography (HPLC)^26^ and spectrophotometry^27^ to analyze the permeation of different drugs through the skin. Contrary to, for example, chromatography, quantitative NMR spectroscopy (qNMR) is directly quantitative since the area under the curve for a resonance signal is directly proportional to the number of nuclei that gives rise to the signal.^28^ Furthermore, as opposed to the traditional usage of Franz diffusion cells, where samples are taken from the receptor chamber at given time intervals and the receptor medium is replaced for every sample, qNMR allows for easy and direct analysis of the precise amount of CBD left in the transdermal patch sample and thus gives a more accurate value of the released amount of CBD from the patch. This is especially true since CBD has affinity for the skin‐mimicking membrane and will accumulate during the experiment. This will render the quantitative release results from the receptor medium unreliable, no matter how precise the analysis of the receptor medium is.

Thus, a combination of Franz cell diffusion with qNMR may be a valuable method to establish and analyze the release and skin permeation of CBD from dermal patches, which has not, to the best of our knowledge, been accurately determined previously. A precise understanding of the release profile and skin permeation of CBD from transdermal patches is essential for broad adaptation of CBD patches since release profiles indicate optimal wear time, reliability, the effectiveness of the matrix, and how the expected effects of CBD may change during the wear time concerning plasma levels.

In this article, we describe a new method for quantitatively determining CBD release from dermal patches using Franz cell diffusion in combination with quantitative ^1^H‐NMR. We tested CBD patches with known CBD content, as well as five different commercially available CBD patches and one market‐ready CBD patch.

## 2 MATERIALS AND METHODS

### 2.1 Materials

The tested transdermal patches represented four different brands, the names of which are available from the author upon request. In this article, they are denoted A, B, C, and D, respectively: Patch A with 40 mg CBD per patch as stated by the manufacturer and described as a cotton/nylon blend with a water‐based adhesive, Patch B with 40 mg CBD per patch as displayed by the manufacturer (no description of patch available), three different compositions from brand C, of 50, 75, and 100 mg CBD, as stated by the manufacturer (no description of patch available), and Patch D with 36 mg CBD as stated by the company (a market‐ready product) described as a glycerol‐silicone adhesive with CBD embedded in the discrete glycerol droplets.

CBD crystals (99% purity) were acquired from ENDOCA (batch No. 1965) and stored in the original glass container under dark conditions at room temperature until use.

The two‐part silicone system was supplied by Wacker Chemie AG, MCT oil (mixture of medium‐chain triglycerides) (food and medical grade, 100%) was obtained from NUTRICIA, glycerol (analytical grade, 99.5%) was obtained from AppliChem GmbH, ethanol absolute (≥99.8%) and CDCl_3_ (for NMR spectroscopy, 99.8% D) were purchased from VWR, Denmark, naphthalene (99%), the Strat‐M™ membrane (Transdermal Diffusion Test Model, 25 mm diameter), polyoxyethylene (20) sorbitan monooleate (TWEEN® 80, emulsifier), and polyethylene glycol 400 (PEG400) were procured from Merck KGaA, Germany, and Pt catalyst, Catalyst 511, platinum content 1.0 wt.%, vinyl content 0.55 mmol/g, was kindly provided by Evonik, Germany. Polyurethane (PU) backing (30 μm thick) was purchased from Transcontinental Advanced Coatings, UK, polyethylene terephthalate (PET) support (75 μm thick) was acquired from HOSTAPHAN®, Mitsubishi Polyester Film, adhesion primer, MED6‐161, was obtained from NuSil, and PET release liner, one surface with fluorosilicone‐based coating (50 μm thick), was purchased from Siliconature S.P.A., Italy. All chemicals were used as received.

### 2.2 Methods

#### 2.2.1 Preparation of patches with known CBD content

Five glycerol‐silicone transdermal patches^29^, ^30^, ^31^ were prepared with known CBD content to verify the qNMR method for CBD quantification. The patches were prepared as follows, with an example of the weighed amounts given for one of the patches in parentheses: CBD (0.37 g) was dissolved in MCT oil (0.11 g) along with surfactants, Tween 80 (0.06 g), and PEG 400 (0.28 g). Glycerol (2.81 g) was then added to the mixture, which was then stirred on a speed mixer (DAC 150.1 FVZ‐K, Synergy Devices Ltd, UK) at 3500 rpm for 3 min. Part A (3.34 g) of the silicone elastomer system was added to the mixture, which was speed‐mixed, whereafter Part B (3.69 g) was added and mixed in using speed mixing. The sample mixture was then coated on a PU backing with primer, prepared as follows: PU backing (30 μm thick) was placed and attached using tape on a PET support (75 μm thick). The PU backing was coated with a thin and uniform layer of the adhesion primer, MED6‐161, using a lint‐free wipe and 1.5 ml of the primer. The primer was allowed to react for 2 h, and excess primer was wiped off. The silicone mixture was coated on the PU backing using an applicator (automatic film coater, 4340, Elcometer, UK) with a gap of 400 μm and a coating speed of 5 mm/s. The samples were cured at 90°C for 30 min, and the cured samples were then applied with PET release liners.

#### 2.2.2 Optical microscopy

The cross‐sectional morphology of the commercial patches, including top release liner, middle adhesive, and bottom substrate, was investigated with a Leica optical microscope (DM LB, Leica Microsystems GmbH, Germany) equipped with a Leica MC190 HD camera at room temperature.

The cross‐section of the patch was vertically cut using a sharp new blade (Azpack™ Carbon Steel Razor Blades, Fisher Scientific, Denmark). Subsequently, the patch was fixed on the vertical side of a microscope slide and with the cut cross‐section horizontally facing up under the lens of the optical microscope.

#### 2.2.3 Franz cell diffusion tests

Five small samples from each of the six commercially available/market‐ready dermal patches were cut as circles, with diameters of 10 mm, corresponding to the size of the hole in the Franz cell (diameter = 10 mm). The CBD patch sample was placed on a transdermal diffusion test model Strat‐M™^32^ membrane. The Strat‐M™ membrane mimics skin conditions of the epidermis and dermis because it consists of two layers of polyether sulfone on top of one layer of polyolefin, which is saturated with synthetic lipids. This creates a porous structure with a gradient in terms of pore size and diffusivity.^27^ The receptor medium in the Franz cell setup (Figure 3) was 7:3 propylene glycol:ethanol (mass ratio), which was previously found to be a well‐suited solvent for CBD.^33^ The rotary speed of the magnetic stir bar was 200 rpm, and the heating temperature was set to 35°C, which corresponds to ~32°C at the membrane (skin temperature). The patches were removed from the setup for ^1^H‐NMR testing after 4, 12, 24, 36, and 48 h. A new 10‐mm sample was used for testing each specific time frame.

#### 2.2.4 Quantitative ^1^H‐NMR

CBD was quantified using qNMR on a Bruker 250 MHz spectrometer. All samples were tested in deuterated chloroform (CDCl_3_)_._ For the verification of the method, CBD and naphthalene were dissolved in CDCl_3_, the ^1^H‐NMR spectra were recorded, and the resonances were identified.

Samples of known CBD content were tested by adding the prepared CBD‐containing patch with known CBD content (see preparation method for patches above) to a vial containing 10 mg naphthalene and 15 ml CDCl_3_, so the patch became completely submerged. A magnet was added, and the screw cap lid was sealed tight, whereafter parafilm was tightly bound around the vial and lid. Aluminum foil was wrapped around the vial to block the light. The vial was stirred on a magnetic stirrer in a dry fume hood for 20 h. Afterward, around 1.5 ml of the solution was transferred to an NMR tube and analyzed by qNMR—five patches were prepared, extracted, and analyzed in duplicate.

For the determination of the total amount of CBD in the commercial/market‐ready dermal patches, each commercial patch was cut into small pieces, which were all placed in a vial with 10 mg of the internal standard, naphthalene, and 15 ml CDCl_3_. The vial was covered with parafilm and aluminum foil to avoid evaporation and light, respectively. The vial was placed on a roller mixer for 2 days at room temperature. At this point, all CBD had diffused into the solvent, CDCl_3_. The CDCl_3_ did not visibly dissolve the parafilm nor were any signals from parafilm detected in the NMR analysis. Approximately 1.5 ml was then transferred to an NMR tube and tested.

During the skin permeation and release studies with Franz cell diffusion, each CBD patch (diameter = 10 mm, area = 79 mm^2^) was added to 1 ml (1.5 g) CDCl_3_ with 1 mg of naphthalene as an internal standard. The ^1^H‐NMR spectra were recorded three times for each sample.

The CBD amount in the patches was thus determined before the Franz cell experiment and after 4, 12, 24, 36, and 48 h in the Franz cell. The CBD amount was calculated by subtracting the original CBD amount in the patch from the calculated amount of CBD in the patch after a given treatment time.

#### 2.2.5 High‐performance liquid chromatography–mass spectroscopy

Quantification of CBD was achieved by HPLC coupled with mass spectroscopy (MS) using an Agilent 1290 Infinity II UHPLC (Agilent Technologies; Santa Clara, CA) equipped with an Agilent Poroshell 120 Phenyl Hexyl column (1.9 μm, 150 × 2.1 mm) coupled to an Agilent 6545 QTOF. Gradient elution was used with eluent A consisting of H_2_O and eluent B consisting of acetonitrile, both of which contained 20 mM formic acid. Gradient elution started at 10% eluent B, increasing to 100% over 10 min, and held at 100% for 2 min, all at a constant flow rate of 0.35 ml/min and a constant column temperature of 60°C. Ionization was achieved using positive electrospray ionization, and mass spectra were obtained with an *m*/*z* range of 100–1,600, acquired at a rate of 10 scans/s. All MS analyses were conducted with purine and HP‐0921 as internal standards for spectrum calibration. The monoisotopic ion mass of the CBD proton adduct was used for integration with a mass accuracy of 6 ppm and a calibration curve made with four levels (0.7, 0.35, 0.175, 0.088 mg/ml). Two replicates of CBD were used to determine the concentration of CBD in samples.

## 3 RESULTS AND DISCUSSION

The release and skin permeation of CBD from transdermal patches were investigated using a new method of Franz cell diffusion in combination with qNMR. The first part of the study consists of a verification of qNMR as a quantification tool for CBD. The second part of the study includes an evaluation of the release of CBD from commercially available/market‐ready transdermal patches.

### 3.1 Verification of qNMR for quantification of CBD in dermal patches

**FIGURE 1:**
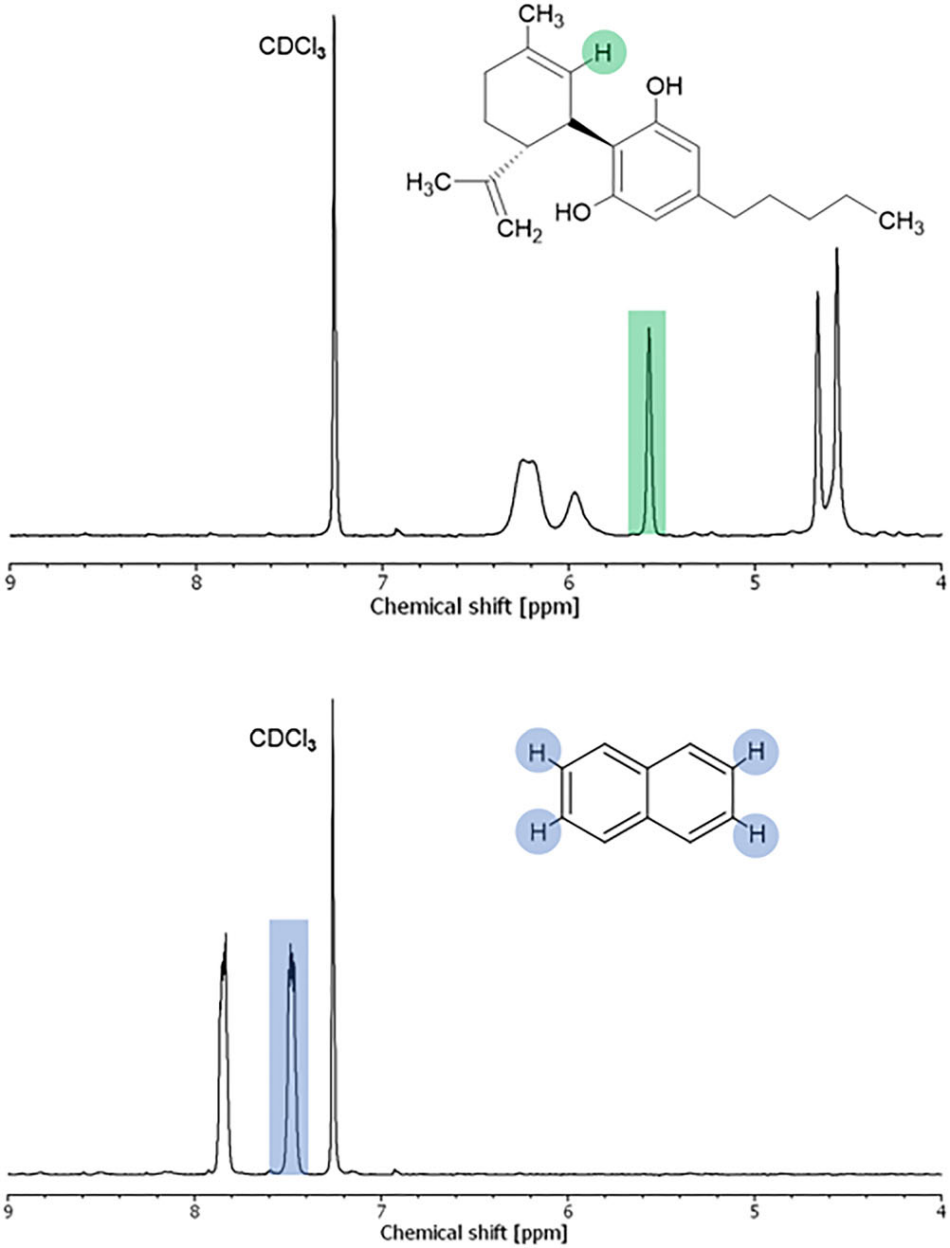
^1^H‐NMR spectra of CBD (top) and naphthalene (bottom) and highlighted protons used for qNMR analysis.

In the first part of the study, the suitability and reliability of qNMR as a quantification method for CBD in dermal patches were determined. Firstly, ^1^H‐NMR spectra of pure CBD and the internal standard (calibrant) naphthalene were recorded in CDCl_3_, where peak resonances were identified. A complete peak identification of CBD^34^ and naphthalene can be found as supporting information, whereas ^1^H‐NMR spectra with the relevant peak resonances for further calculations highlighted are shown in Figure 1.

Secondly, samples with known amounts of CBD were prepared and analyzed to verify the reliability of the developed qNMR method. Five two‐phase glycerol‐silicone patches with a known content of CBD varying from 33 to 43 mg were prepared and analyzed by ^1^H‐NMR in duplicate. These transdermal patches were based on hybrid drug delivery elastomers previously described in Mazurek et al.^29^, ^35^ The content of CBD in the prepared samples was then calculated as follows:

(1) $$m_{\mathrm{CBD}}=\frac{I_{\mathrm{CBD}}}{I_{\mathrm{cal}}}\times \frac{N_{\mathrm{cal}}}{N_{\mathrm{CBD}}}\times \frac{m_{\mathrm{cal}}}{{\mathrm{MW}}_{\mathrm{cal}}}\times {\mathrm{MW}}_{\mathrm{CBD}}\times \frac{p_{\mathrm{cal}}}{p_{\mathrm{CBD}}},$$

where *m*_*CBD*_ is the content of CBD in mg, *I*_*CBD*_ is the integral of the CBD peak at 5.57 ppm (peak highlighted in the CBD spectrum in Figure 1), *I*_*cal*_ is the integral of the calibrant (naphthalene) peak at 7.48 ppm (peak highlighted in the naphthalene spectrum in Figure 1), *N* is the number of nuclei assigned to each peak (*N*_*cal*_/*N*_*CBD*_ = 4/1 = 4), *m*_*ca*l_ is the known mass of naphthalene in mg, *MW*_*cal*_ is the molecular weight of naphthalene (128.17 g mol^−1^), *MW*_*CBD*_ is the molecular weight of CBD (314.47 g mol^−1^), *p*_*cal*_ is the purity of the calibrant naphthalene (0.99), and *p*_*CBD*_ is the purity of CBD (0.99). In this instance, the purity is stated by the manufacturer; if the purity is unknown, it can be determined by analytical methods such as spectroscopy and chromatography. The results of the qNMR testing of the five prepared samples, in the form of peak integral ratios, *I*_*CBD*_/*I*_*cal*_, are shown in Table 1, along with the calculated masses of CBD as well as the known mass of CBD and the resulting percentage of recovery (*m*_*CBD*_ *calculated*/*m*_*CBD*_ *in sample* × 100%).

**TABLE 1:** Results of the qNMR testing, for samples with known CBD content, in the form of peak integral ratios, *I*_*CBD*_/*I*_*cal*_, as well as the calculated mass of CBD, the known mass of CBD, and the percentage of the recovery

Sample No.	*I*_*CBD*_/*I*_*cal*_	*m*_*CBD*_ *calculated* [mg]	*m*_*CBD*_ *in sample* [mg]	Recovery [%]
1	0.137	40.9	43.1	94.9
2	0.342	35.2	33.3	105.7
3	0.408	40.4	36.6	110.6
4	0.343	34.0	35.9	94.7
5	0.328	32.5	33.8	96.1
**Total**				100.4 ± 7.3

As shown in Table 1, the obtained percentages of recovery using qNMR as a quantification tool for determining the content of CBD in transdermal patches vary from 94.7% to 110.6% for the five tested samples with known CBD content. This yields an average recovery of 100% ± 7%. The slight deviations observed could, in principle, be due to variations in CBD concentration across the patch and not the qNMR analysis method since a small sample of 10 mm is cut from a larger patch for analysis. The results, however, verify that the process of extraction, as well as the method of quantification using qNMR, is a reliable methodology for analyzing CBD in transdermal patches. For comparison, the recovery of CBD using quantitative HPLC has previously been shown to lie in the range of 85–90%.^36^, ^37^ To further compare qNMR with quantitative HPLC, six identical glycerol‐silicone samples with known CBD content were tested using HPLC‐MS and qNMR. The results are presented in detail in the supporting information. qNMR yielded an average recovery of 98.4% ± 2% for these specific samples, whereas HPLC‐MS yielded an average recovery of 94.2% ± 2%. Loss of CBD during the filtration step before the HPLC‐MS measurements may be responsible for the deviation between the two analysis methods.

### 3.2 Skin permeation and release of CBD from dermal patches

**FIGURE 2:**
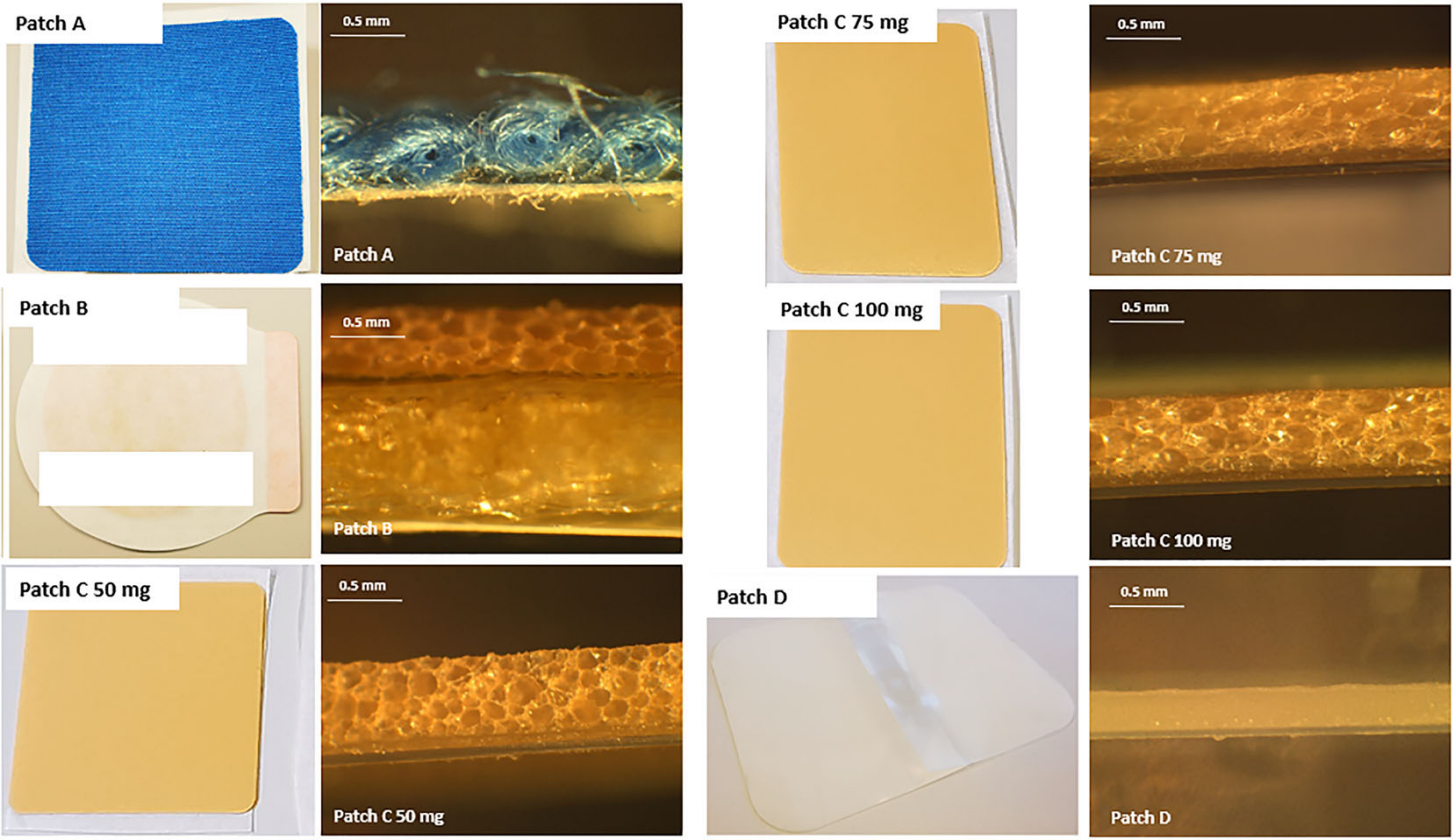
Images and cross‐sectional images of the six tested commercially available/market‐ready patches from four brands.

In the second part of the study, the skin permeation and release of CBD from dermal patches were determined using Franz cell diffusion tests in combination with qNMR. Six different patches from four brands were tested, namely Patch A with 40 mg CBD per patch as stated by the manufacturer, Patch B with 40 mg CBD per patch as stated by the manufacturer, three different patches of brand C with 50, 75, and 100 mg CBD, as stated by the manufacturer, and Patch D with 36 mg CBD as stated by the company. Photos of the patches, as well as cross‐sectional images, are shown in Figure 2.

The starting amounts of CBD in the dermal patches of interest, A, B, C 50 mg, C 75 mg, C 100 mg, and D, were determined by qNMR in triplicate and averaged. Representative resulting ^1^H‐NMR spectra of dermal patches in CDCl_3_ with naphthalene as an internal standard can be found in the electronic supporting information (ESI). The content of CBD in the dermal patches was then calculated according to equation 1. The results of the qNMR testing of the dermal patches, in the form of peak integral ratios, *I*_*CBD*_/*I*_*cal*_, are shown in Table 2. The calculated content of CBD (*m*_*CBD*_) is furthermore presented in Table 2, as well as the difference between the calculated CBD content and the content stated by the manufacturer ((│*m*_*CBD*_ *calculated* − *m*_*CBD*_ *labeled*│)/((*m*_*CBD*_ *calculated* − *m*_*CBD*_ *labeled*)/2) × 100%).

**TABLE 2:** Results of the qNMR testing in the form of peak integral ratios, *I*_*CBD*_/*I*_*cal*_, of CBD dermal patches before the Franz cell diffusion test (starting CBD amounts), the determined amount of CBD, and the amount of CBD stated by the manufacturer/company, as well as the area and thickness of the patches

Brand	Adhesive thickness [μm]	Patch area [cm^2^]	*I*_*CBD*_/*I*_*cal*_	*m*_*CBD*_ determined [mg]	*m*_*CBD*_ stated by manufacturer/company [mg]	Difference between labeled and determined value [%]
A	90	52.4	0.042 ± 0.004	27.7	40	−36
B	900	4.91	0.407 ± 0.027	45.9	40	14
C 50 mg	75	25.5	0.243 ± 0.008	67.8	50	30
C 75 mg	75	50.4	0.166 ± 0.011	104.3	75	33
C 100 mg	56	50.4	0.248 ± 0.041	135.3	100	30
D	250	41.9	0.069 ± 0.002	36.0	36	0

From the results in Table 2, it is clear that the labeled amount in most of the transdermal patches does not align with the amount found via qNMR. Since the verification process proved that it was possible to account for almost the exact amount of CBD in the laboratory‐made patches with known CBD content, it is reasonable to conclude that the investigated transdermal CBD patches are not labeled accurately according to their actual content. HPLC‐MS analyses displayed similar correlations as the qNMR results when considering the potential deviation from the filtration step. The results of HPLC‐MS analyses on the commercial patches can be found in the supporting information.

The difference between stated content and the actual content in CBD products has also previously been described by others, including Bonn‐Miller et al.,^38^ who found that only 31% out of 84 CBD products were labeled correctly. The analyses were performed using HPLC. According to standards from medicinal cannabis leaders, a ±10% variance in CBD content is considered an acceptable margin for correct labeling of products.^38^ The results presented here add to the growing evidence^39^, ^40^, ^41^, ^42^ that online CBD products have a high rate of mislabeling and that there is a need for regulations and good manufacturing practices. Under‐labeled products, for example, pose the risk of adverse effects since drug–drug interactions are probable and impact serum levels when other medications are simultaneously administered to patients.^39^ Out of the six tested CBD patches in this study, only one patch, namely Patch D, was correctly labeled according to the ±10% variance margin, whereas four were under‐labeled and one was over‐labeled. In the case of Patch A, where a significantly lower amount (−36%) of CBD was recovered compared to the CBD content stated by the manufacturer, it, however, must be considered that the matrix material of this patch (see Figure 2) does not allow for complete release of CBD and that some CBD remains undetected.

**FIGURE 3:**
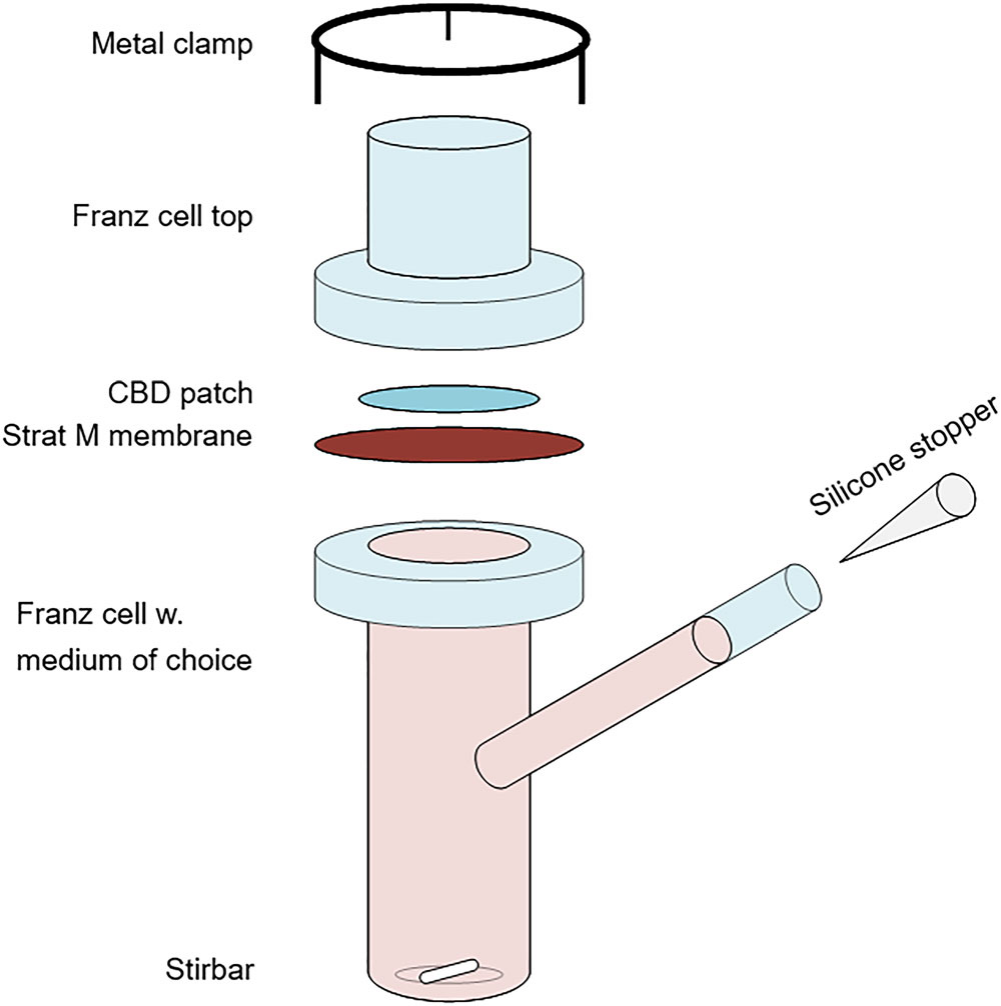
Schematic illustration of a static Franz cell diffusion setup for in vitro transdermal diffusion studies.

The skin permeation and release of CBD from the different transdermal patches were analyzed in vitro by placing a small sample with a diameter of 10 mm from each commercial CBD dermal patch into the Franz cell diffusion setup depicted in Figure 3 on a skin‐mimicking Strat‐M™^32^ membrane.

**FIGURE 4:**
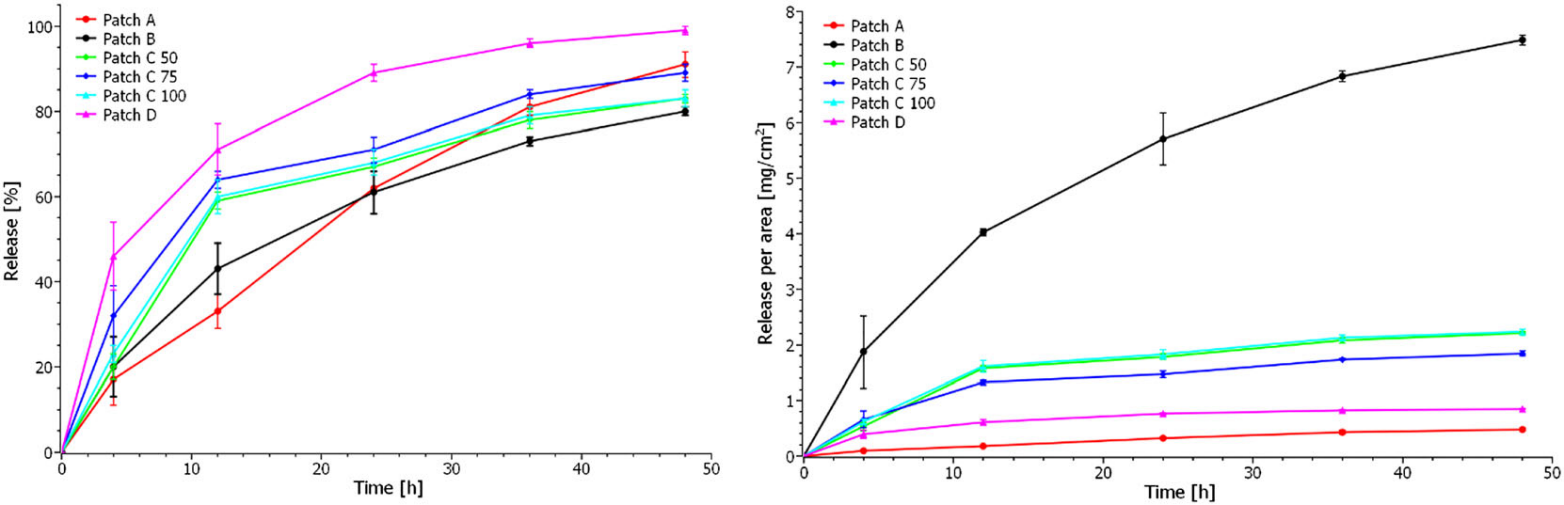
Release profiles in the form of a percentage of release as a function of time (left) and release per area as a function of time (right) for release of CBD from commercial patches as determined from Franz cell diffusion in combination with qNMR.

Each commercial CBD patch was analyzed by qNMR after 4, 12, 24, 36, and 48 h in the Franz cell, whereafter the remaining CBD content in the patches in mg was calculated using equation 1. The obtained release profiles in the form of release percentage as a function of time and release per area as a function of time are shown in Figure 4. The release percentage was calculated as *m*_*CBD*_ *calculated*/*m*_*CBD*_ *total (qNMR)* × 100%, where *m*_*CBD*_ *total (qNMR)* is the starting amount of CBD in each commercial patch as determined by qNMR and listed in Table 2. The release per area (mg/cm^2^) was calculated as *m*_*CBD*_ calculated/patch area, where the patch area is listed in Table 2.

Figure 4 shows that the release of CBD after 4 h varies from 17% ± 6% for Patch A to 46% ± 8% for Patch D, which thus has the highest initial release of CBD. This is, however, not indicative of burst release (rapid release of substances at the beginning of the release process) but follows release profiles for similar matrices with active substances.^31^ Furthermore, Patch D is made from silicone, which swells in the receptor medium, so most likely, a more significant fraction of CBD is initially extracted from this patch compared to the other patches.

After 48 h, the patches had released between 80% ± 1% (patch B) and 99 ± 1% (Patch D) of their embedded CBD.

The release per area as a function of time reveals that Patch B releases large amounts of CBD per area because the patch is significantly smaller than the other patches. It will thus give a high concentration of CBD to a smaller area on the skin. Efficient skin permeation into the dermis is thus of great importance for this patch type to avoid skin irritation.

**FIGURE 5:**
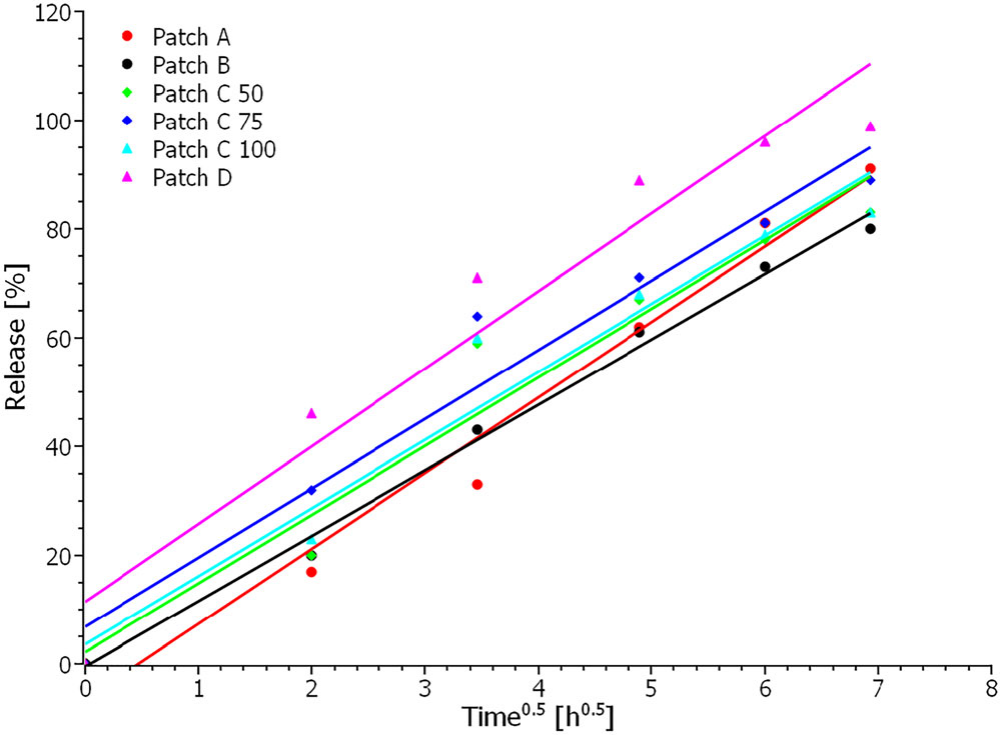
CBD release from transdermal patches plotted as release% as a function of *t* ^0.5^ according to the Higuchi model.

The release of CBD from the various commercial patches was evaluated and compared via drug release kinetics. Table 3 illustrates the most commonly used drug release kinetic models and the resulting squared correlation coefficient (r^2^), obtained by applying the different models to the data. Zero‐order kinetics describe a constant release of a drug that is not concentration‐dependent, first‐order kinetics describe drug release which is dependent on concentration, and the Higuchi model describes drug release as a diffusion process based on Fick's law which is square root time‐dependent. In contrast, the Weibull function may describe more complex release mechanisms, where the shape parameter's value, β, indicates the transport mechanism.^43^ The model that best fits the release data is evaluated by comparing the squared correlation coefficients. The model that yields the highest coefficient can be said to fit the data best. Overall, the obtained release data for all transdermal patches is best described by the Higuchi model (r^2^ > 0.9 for all patches); however, the Weibull model is equally suited to describe the release from Patch D. The release profiles using the Higuchi model are shown in Figure 5. From these plots, the release rate as release%/*t* ^0.5^ was calculated and utilized to compare the release from the different patches with the results shown in Table 3. From Table 3 and Figure 5, it is clear that the highest release rates are obtained for Patches A and D, whereas the lowest release rates are obtained for Patches C and B. The three C patches have similar release rates independent of their initial CBD content. Furthermore, these patches and Patch B have similar morphologies as seen from the cross‐sectional images in Figure 2, perhaps leading to similar release rates of CBD. However, obtaining any specific information on the matrix material of these patches was not possible. The manufacturer states that Patch A consists of a textile matrix, which is also evident from Figure 2, while Patch D is a glycerol‐silicone type adhesive. The release rate is seemingly independent of starting content of CBD in the patches. Thus, it is reasonable to assume that the matrix of the patches plays a crucial role in the diffusivity of CBD. Consequently, the obtained release rate with the Higuchi model confirms that the release is diffusion‐controlled.

**TABLE 3:** The most commonly used drug release kinetic models and the resulting squared correlations coefficient, r^2^, as well as the calculated release rates and extrapolated time of 100% release

Model	Equation	Graphical representation	r^2^					
			A	B	C 50	C 75	C 100	D
Zero‐order	$Q_{t}=Q_{0}+K_{0}t$	Release% vs. *t*	0.97	0.90	0.81	0.79	0.80	0.74
First order	$Q_{t}=Q_{0}e^{-\mathrm{Kt}}$	Log(release%) vs. *t*	0.63	0.55	0.52	0.46	0.50	0.42
Higuchi	$Q_{t}=K_{H}t^{0.5}$	Release% vs. *t* ^0.5^	0.98	0.99	0.95	0.95	0.95	0.94
Weibull	$Q_{t}=\left(100-Q_{0}\right)\left[1-e^{-{\mathrm{\alpha t}}^{\beta }}\right]$	Log(release%) vs. log(time)	0.93	0.89	0.87	0.82	0.86	0.95
**Release rate based on Higuchi**			**[release%/*t*** ^**0.5**^ **]**					
Release rate			13.9	12.0	12.6	12.7	12.5	14.3
**Extrapolated time of 100% release**			**[h]**					
Time			59	70	60	54	59	39

Here, *Q*_0_ is the initial percentage of the drug at time *t* = 0, *Q*_*t*_ is the percentage of drug released at time *t*, *K*_0_ is the zero‐order release constant, *t* is time in h, *K* is the first‐order release constant, *K*_*H*_ is the Higuchi constant, β is the Weibull shape parameter, and α is the Weibull scale factor.

When using the Higuchi model to extrapolate the data to 100% release, assuming the release trends will continue for all patches, it is possible to predict the time point when each patch will have released all its CBD content. The results of the extrapolation are shown in Table 3, from which it can be seen that Patch D has released all CBD after approximately 39 h. In contrast, Patch A is predicted to have released all CBD after about 59 h, Patch B after 70 h, Patch C 50 mg after 60 h, Patch C 75 mg after 54 h, and Patch C 100 mg after 59 h.

It is evident from the obtained release data, that the 6 different commercial CBD patches have different release profiles and thereby quite different skin permeation profiles with a variation of around 30 hours from the depletion of the fastest releasing patch to the depletion of the slowest releasing patch. This means that the plasma levels of CBD will most likely be very different when applying the various CBD patches. The new methodology, Franz cell diffusion in combination with qNMR, of determining the release from CBD patches can thus be an essential tool for selecting the most appropriate CBD patch for specific uses and medical conditions and establishing the effectiveness and reliability of CBD dermal patches.

## CONFLICTS OF INTEREST

Anne Ladegaard Skov has, as the CSO of brand D, commercial interests in one of the tested CBD patches.

## Supporting information


**Figure S1:** ^1^H‐NMR spectrum of pure CBD with the chemical structure of CBD shown along with the numbers used for peak identification.
**Figure S2:** ^1^H‐NMR spectrum of pure naphthalene with the chemical structure shown along with the numbers used for peak identification.
**Figure S3:** Representative ^1^H‐NMR spectrum of patch A in CDCl_3_ with naphthalene as calibrant.
**Figure S4:** Representative ^1^H‐NMR spectrum of patch B in CDCl_3_ with naphthalene as calibrant.
**Figure S5:** Representative ^1^H‐NMR spectrum of patch C 50 mg patch in CDCl_3_ with naphthalene as calibrant.
**Figure S6:** Representative ^1^H‐NMR spectrum of patch C 75 mg patch in CDCl_3_ with naphthalene as calibrant.
**Figure S7:** Representative ^1^H‐NMR spectrum of patch C 100 mg patch in CDCl_3_ with naphthalene as calibrant.
**Figure S8:** Representative ^1^H‐NMR spectrum of patch D in CDCl_3_ with naphthalene as calibrant.
**Table S1.** Results of the qNMR and HPLC‐MS testing, for six samples with known CBD content, in the form of the percentage of the recovery.
**Table S2.** Results of the HPLC‐MS testing in the form of the determined amount of CBD and the CBD stated by the manufacturer/company as well as the difference between the labeled and determined amount.

## Data Availability

The original data can be obtained by writing to the corresponding author by email.
