# Supplementary material for: A reliable quantitative method for determining CBD content and release from transdermal patches in Franz cells
Source: Phytochem Anal. 2022 Nov 13;33(8):1257–65. doi: 10.1002/pca.3188 (PMC10100468; doi:10.1002/pca.3188)
Supplement: Supplementary file 1 — Figure S1: 1H‐NMR spectrum of pure CBD with the chemical structure of CBD shown along with the numbers used for peak identification. Figure S2: 1H‐NMR spectrum of pure naphthalene with the chemical structure shown along with the numbers used for peak identification. Figure S3: Representative 1H‐NMR spectrum of patch A in CDCl3 with naphthalene as calibrant. Figure S4: Representative 1H‐NMR spectrum of patch B in CDCl3 with naphthalene as calibrant. Figure S5: Representative 1H‐NMR spectrum of patch C 50 mg patch in CDCl3 with naphthalene as calibrant. Figure S6: Representative 1H‐NMR spectrum of patch C 75 mg patch in CDCl3 with naphthalene as calibrant. Figure S7: Representative 1H‐NMR spectrum of patch C 100 mg patch in CDCl3 with naphthalene as calibrant. Figure S8: Representative 1H‐NMR spectrum of patch D in CDCl3 with naphthalene as calibrant. Table S1. Results of the qNMR and HPLC‐MS testing, for six samples with known CBD content, in the form of the percentage of the recovery. Table S2. Results of the HPLC‐MS testing in the form of the determined amount of CBD and the CBD stated by the manufacturer/company as well as the difference between the labeled and determined amount. [file PCA-33-1257-s001.docx]

Supporting information

A reliable quantitative method for determining CBD content and release from transdermal patches in Franz cells

# Liyun Yu^1^, Frederikke Bahrt Madsen^1,2^, Sofie Helvig Eriksen^1^, Aaron JC Andersen^3^, and Anne Ladegaard Skov^1,2^

^1^Danish Polymer Centre, Department of Chemical and Biochemical Engineering, Building 227, Technical University of Denmark, DK-2800 Kgs. Lyngby, Denmark

^2^Glysious, Kong Valdemarsvej 58, DK-2840 Holte, Denmark

^3^Department of Biotechnology and Biomedicine, Building 221, Technical University of Denmark, DK-2800 Kgs. Lyngby, Denmark


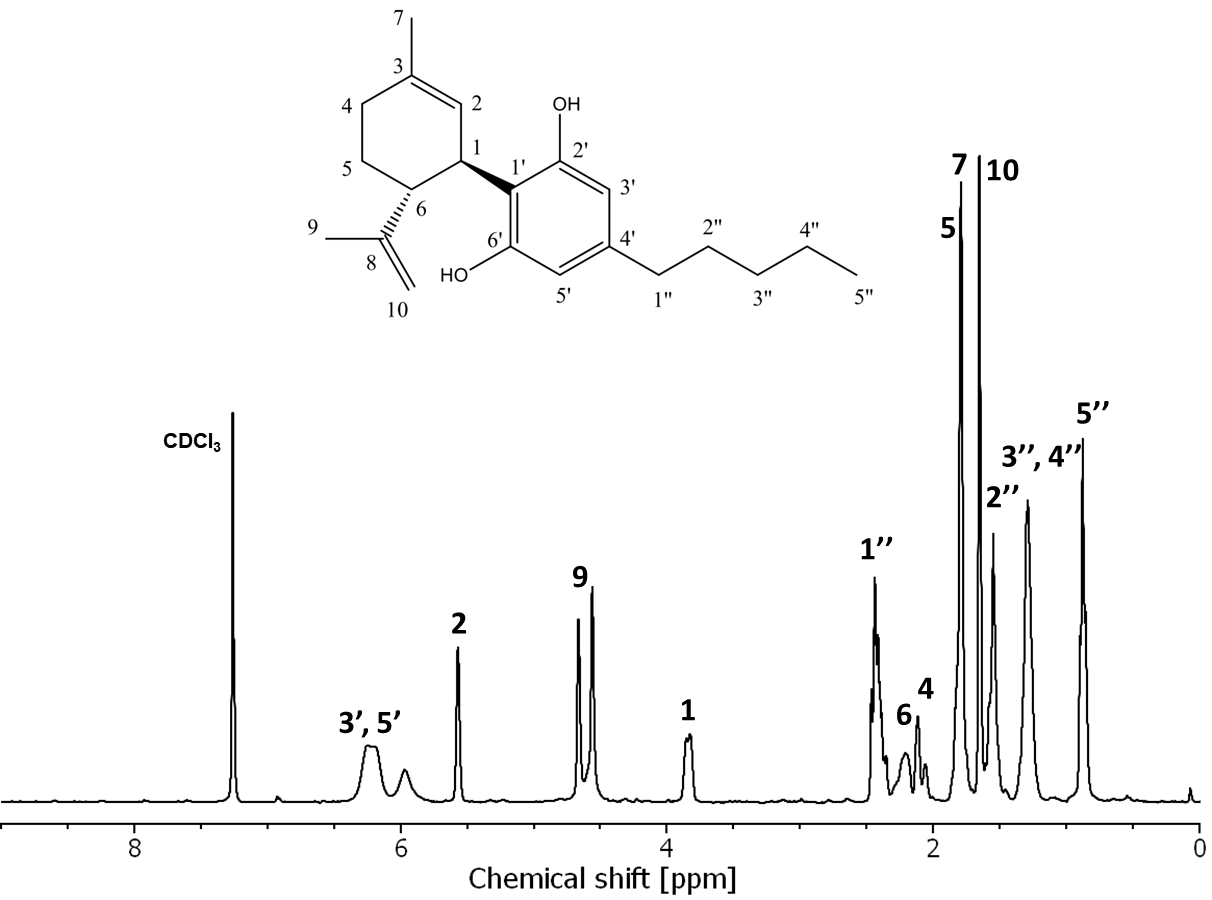


Figure S1: ^1^H-NMR spectrum of pure CBD with the chemical structure of CBD shown along with the numbers used for peak identification.


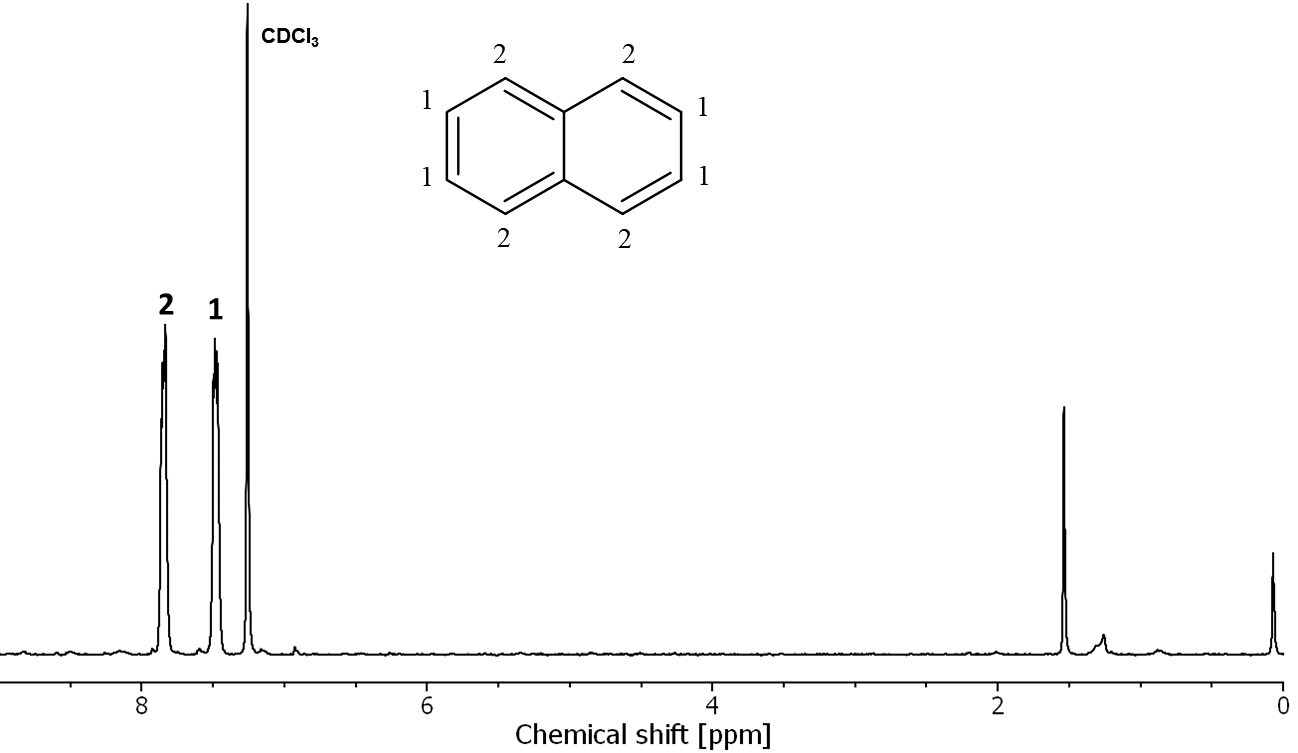


Figure S2: ^1^H-NMR spectrum of pure naphthalene with the chemical structure shown along with the numbers used for peak identification.


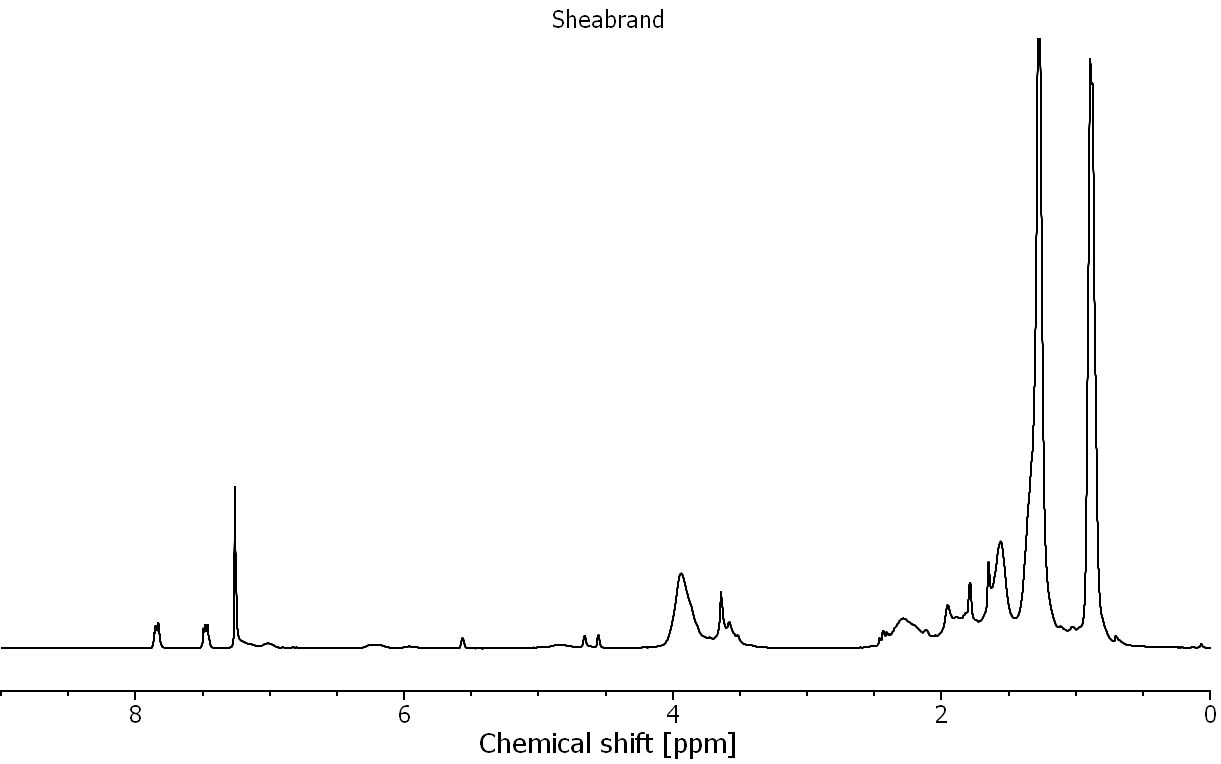


Figure S3: Representative ^1^H-NMR spectrum of patch A in CDCl_3_ with naphthalene as calibrant.


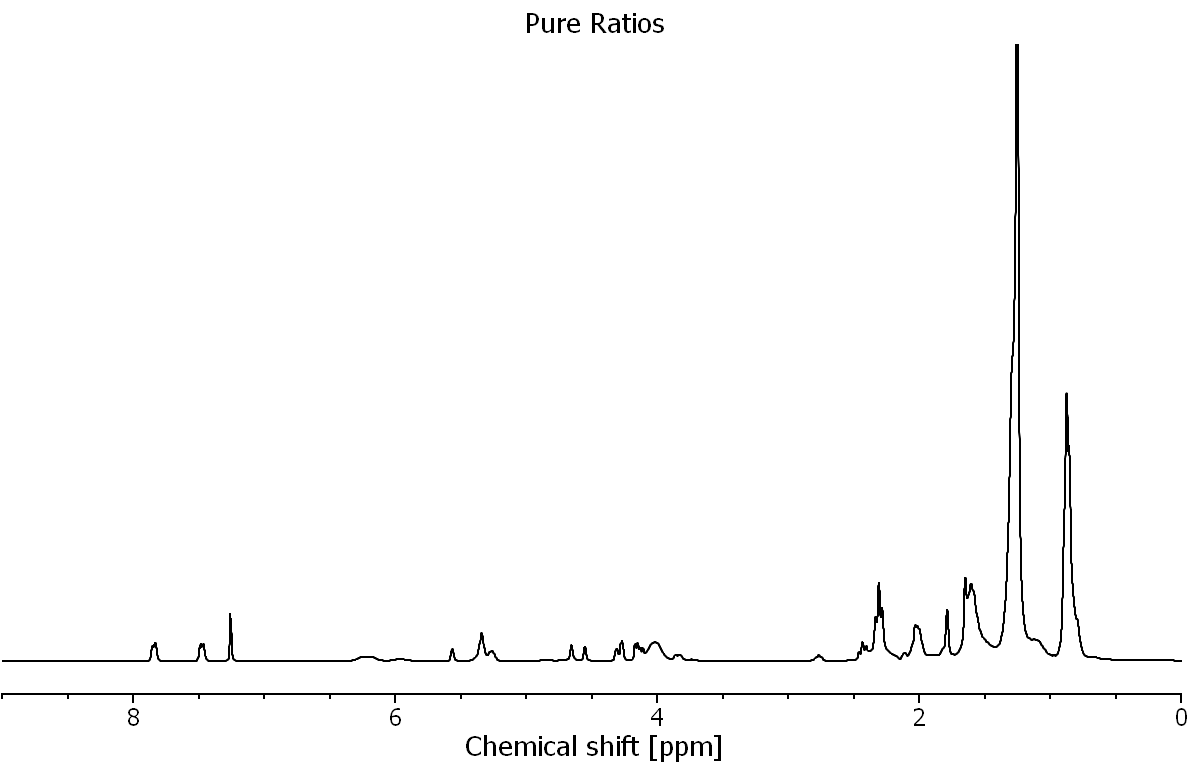


Figure S4: Representative ^1^H-NMR spectrum of patch B in CDCl_3_ with naphthalene as calibrant.


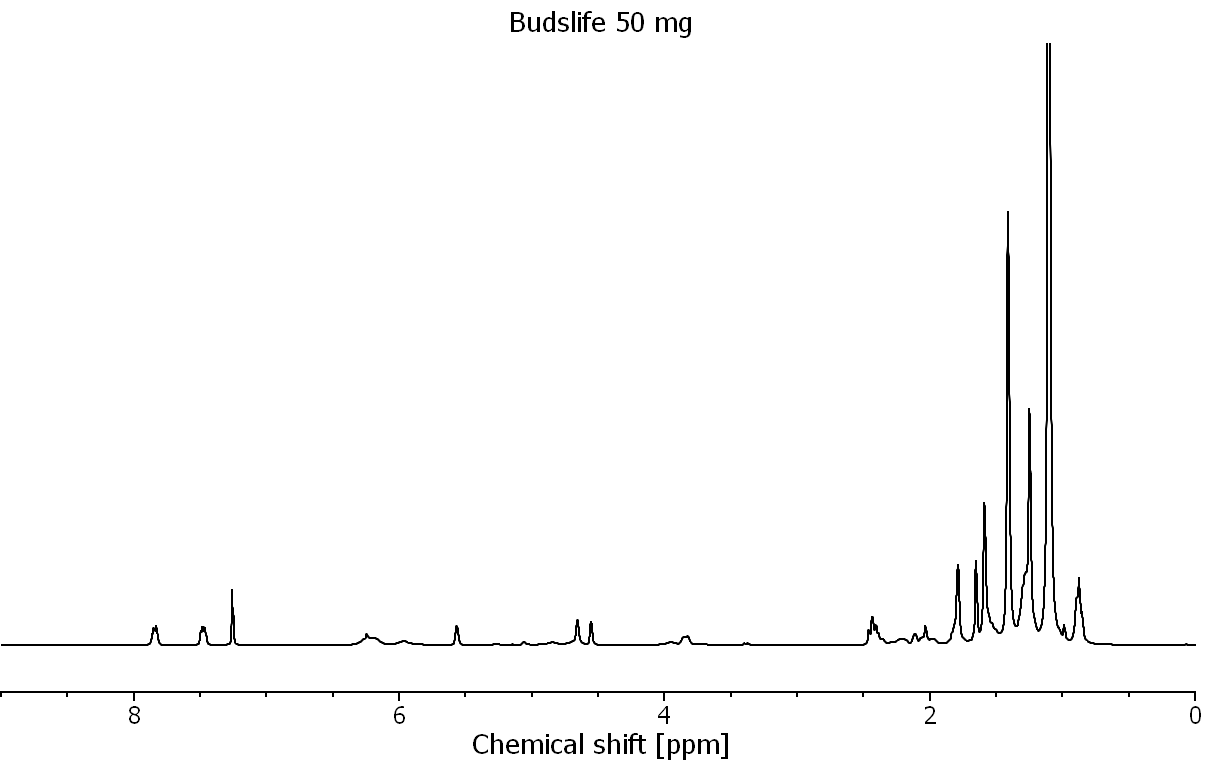


Figure S5: Representative ^1^H-NMR spectrum of patch C 50 mg patch in CDCl_3_ with naphthalene as calibrant.


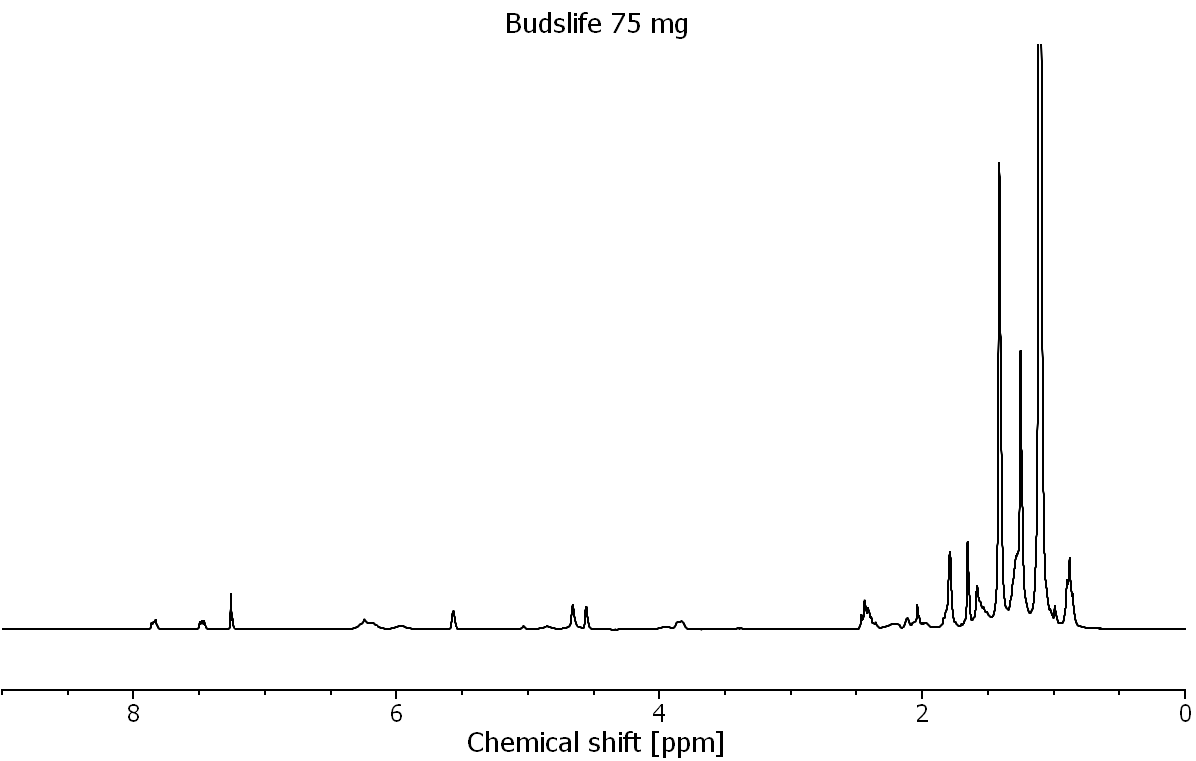


Figure S6: Representative ^1^H-NMR spectrum of patch C 75 mg patch in CDCl_3_ with naphthalene as calibrant.


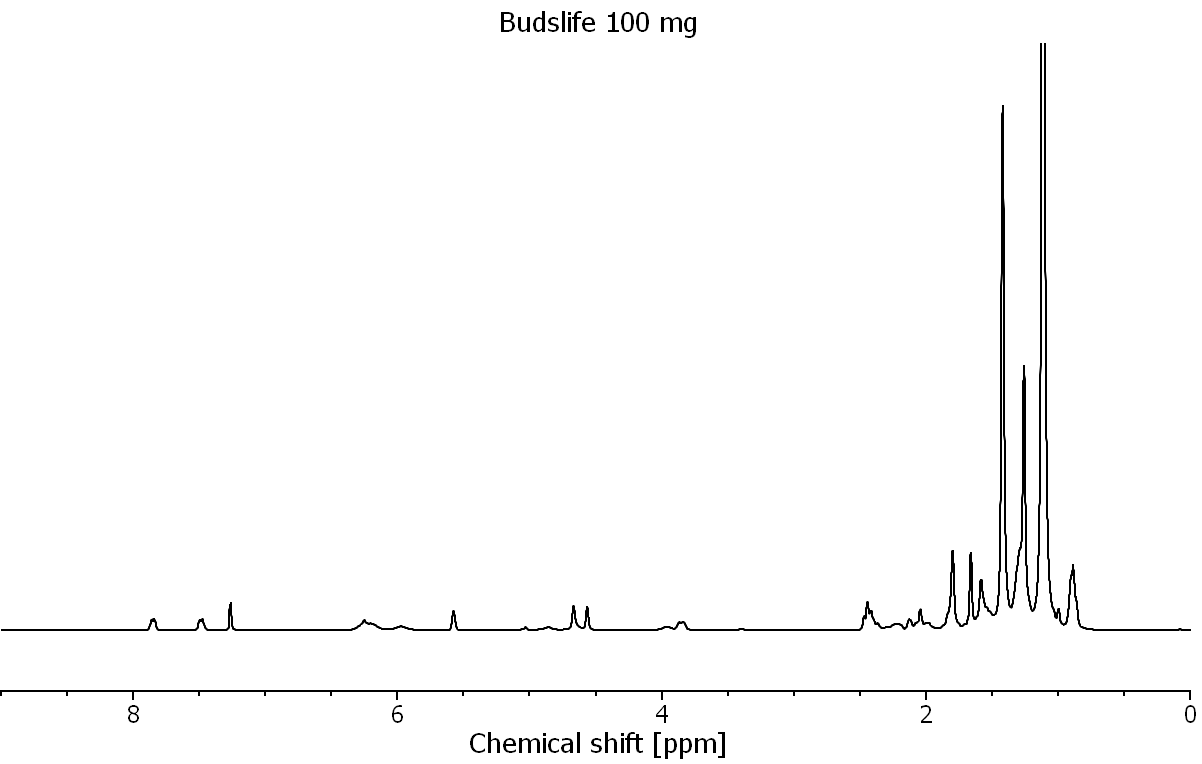


Figure S7: Representative ^1^H-NMR spectrum of patch C 100 mg patch in CDCl_3_ with naphthalene as calibrant.


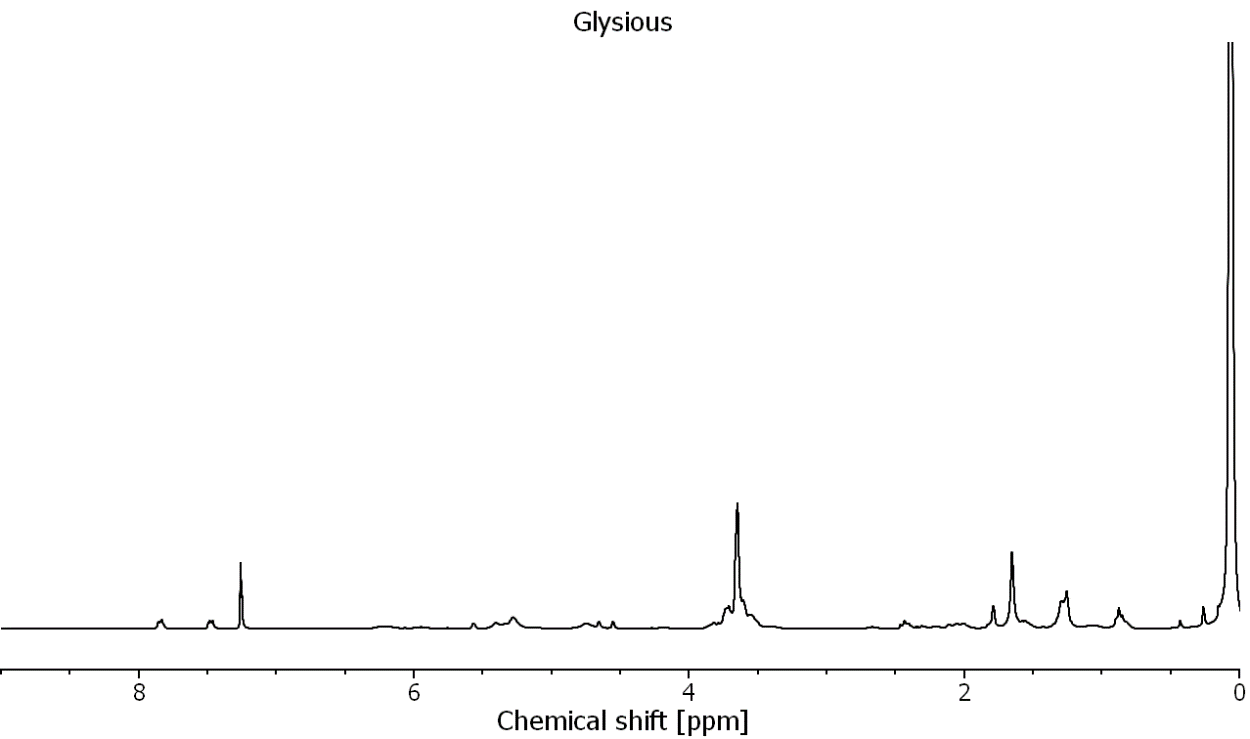


Figure S8: Representative ^1^H-NMR spectrum of patch D in CDCl_3_ with naphthalene as calibrant.

Table S1. Results of the qNMR and HPLC-MS testing, for six samples with known CBD content, in the form of the percentage of the recovery.

| **Sample no.** | **Recovery from qNMR [%]** | **Recovery from HPLC [%]** |
| --- | --- | --- |
| 1 | 100.7 | 96.5 |
| 2 | 95.8 | 96.4 |
| 3 | 99.6 | 91.5 |
| 4 | 99.0 | 95.8 |
| 5 | 99.6 | 92.4 |
| 6 | 95.9 | 92.7 |
| **Total average** | **98.4±2** | **94.2±2** |

Table S2. Results of the HPLC-MS testing in the form of the determined amount of CBD and the CBD stated by the manufacturer/company as well as the difference between the labeled and determined amount.

| **Brand** | ***m_CBD_* determined HPLC-MS [mg]** | ***m_CBD_* stated by manufacturer/company [mg]** | **Difference between labeled and determined value [%]** |
| --- | --- | --- | --- |
| A | 29.3 | 40 | -31 |
| B | 37.2 | 40 | -7 |
| C 50 mg | 61.3 | 50 | 20 |
| C 75 mg | 98.5 | 75 | 27 |
| C 100 mg | 133.1 | 100 | 28 |
